# Subjective cognitive decline and anxious/depressive symptoms during the COVID-19 pandemic: what is the role of stress perception, stress resilience, and β-amyloid?

**DOI:** 10.1186/s13195-022-01068-7

**Published:** 2022-09-06

**Authors:** Muge Akinci, Gonzalo Sánchez-Benavides, Anna Brugulat-Serrat, Cleofé Peña-Gómez, Eleni Palpatzis, Mahnaz Shekari, Carme Deulofeu, Sherezade Fuentes-Julian, Gemma Salvadó, José Maria González-de-Echávarri, Marc Suárez-Calvet, Carolina Minguillón, Karine Fauria, José Luis Molinuevo, Juan Domingo Gispert, Oriol Grau-Rivera, Eider M. Arenaza-Urquijo, for the ALFA Study, Annabella Beteta, Raffaele Cacciaglia, Alba Cañas, Irene Cumplido, Ruth Dominguez, Maria Emilio, Carles Falcon, Laura Hernandez, Gema Huesa, Jordi Huguet, Paula Marne, Tania Menchón, Grégory Operto, Albina Polo, Blanca Rodríguez-Fernández, Sandra Pradas, Iman Sadeghi, Anna Soteras, Laura Stankeviciute, Marc Vilanova, Natalia Vilor-Tejedor

**Affiliations:** 1 Barcelonaβeta Brain Research Center (BBRC), Pasqual Maragall Foundation, Barcelona, Spain; 2 Universitat Pompeu Fabra, Barcelona, Spain; 3 IMIM (Hospital del Mar Medical Research Institute), Barcelona, Spain; 4 Centro de Investigación Biomédica en Red de Fragilidad y Envejecimiento Saludable (CIBERFES), Madrid, Spain; 5 Global Brain Health Institute, University of California San Francisco, San Francisco, CA USA; 6 Department of Clinical Sciences, Clinical Memory Research Unit, Malmö, Sweden; 7 Servei de Neurología, Hospital del Mar, Barcelona, Spain; 8 H.Lundbeck A/s, Copenhagen, Denmark; 9 Centro de Investigación Biomédica en Red de Bioingeniería, Biomateriales y Nanomedicina (CIBER-BBN), Madrid, Spain

**Keywords:** Subjective cognitive decline, Stress, Anxiety, Depression, COVID-19 confinement, Alzheimer’s disease

## Abstract

**Background:**

The COVID-19 pandemic may worsen the mental health of people reporting subjective cognitive decline (SCD) and therefore their clinical prognosis. We aimed to investigate the association between the intensity of SCD and anxious/depressive symptoms during confinement and the underlying mechanisms.

**Methods:**

Two hundred fifty cognitively unimpaired participants completed the Hospital Anxiety and Depression Scale (HADS) and SCD-Questionnaire (SCD-Q) and underwent amyloid-β positron emission tomography imaging with [^18^F] flutemetamol (*N* = 205) on average 2.4 (± 0.8) years before the COVID-19 confinement. During the confinement, participants completed the HADS, Perceived Stress Scale (PSS), Brief Resilience Scale (BRS), and an ad hoc questionnaire on worries (access to primary products, self-protection materials, economic situation) and lifestyle changes (sleep duration, sleep quality, eating habits). We investigated stress-related measurements, worries, and lifestyle changes in relation to SCD. We then conducted an analysis of covariance to investigate the association of SCD-Q with HADS scores during the confinement while controlling for pre-confinement anxiety/depression scores and demographics. Furthermore, we introduced amyloid-β positivity, PSS, and BRS in the models and performed mediation analyses to explore the mechanisms explaining the association between SCD and anxiety/depression.

**Results:**

In the whole sample, the average SCD-Q score was 4.1 (± 4.4); 70 (28%) participants were classified as SCD, and 26 (12.7%) were amyloid-β-positive. During the confinement, participants reporting SCD showed higher PSS (*p* = 0.035) but not BRS scores (*p* = 0.65) than those that did not report SCD. No differences in worries or lifestyle changes were observed. Higher SCD-Q scores showed an association with greater anxiety/depression scores irrespective of pre-confinement anxiety/depression levels (*p* = 0.002). This association was not significant after introducing amyloid-β positivity and stress-related variables in the model (*p* = 0.069). Amyloid-β positivity and PSS were associated with greater HADS irrespective of pre-confinement anxiety/depression scores (*p* = 0.023; *p* < 0.001). The association of SCD-Q with HADS was mediated by PSS (*p* = 0.01).

**Conclusions:**

Higher intensity of SCD, amyloid-β positivity, and stress perception showed independent associations with anxious/depressive symptoms during the COVID-19 confinement irrespective of pre-confinement anxiety/depression levels. The association of SCD intensity with anxiety/depression was mediated by stress perception, suggesting stress regulation as a potential intervention to reduce affective symptomatology in the SCD population in the face of stressors.

**Supplementary Information:**

The online version contains supplementary material available at 10.1186/s13195-022-01068-7.

## Background

The COVID-19 pandemic and subsequent measures implemented to prevent the spread of the disease, such as home confinement, are having a global impact on the mental health of the general population [1, 2]. Several studies conducted during this period indicated that the mental health burden of the pandemic may be greater in clinically vulnerable populations [3–5] such as those with subjective cognitive decline (SCD) [6, 7].

During the preclinical stage, Alzheimer’s disease (AD) pathologies—amyloid beta (Aβ) and tau—slowly aggregate in the brain and interact with other risk factors including anxiety and depression [8–10], eventually leading to cognitive decline and dementia [11]. Before the onset of the cognitive impairment, some individuals may experience SCD defined as the perception of cognitive decline despite having objectively normal performance in neuropsychological testing [12, 13]. SCD is associated with a higher risk of developing AD [14, 15] as well as with the earliest AD-related pathophysiological processes such as Aβ accumulation [14, 16, 17].

Affective symptoms have been linked with an elevated risk for developing AD in cognitively unimpaired (CU) adults [8, 9, 18]. Moreover, growing evidence suggests associations between anxious/depressive symptoms and biomarkers of AD in CU older adults [19–21], such as with brain Aβ burden [22, 23]. These symptoms may also underlie SCD and/or interact with it [24]. Several studies reported that anxiety, depression, and worries are among the most common concurrent affective symptoms with SCD in older adults [24–26]. Moreover, the co-occurrence of SCD with these symptoms has been linked with an increased likelihood of cognitive decline [27–31].

Recent studies found associations between subjective memory complaints and stressful life events in CU older adults [32, 33]. COVID-19-related home confinement, as one of the main stressors related to the pandemic [33, 34], has been related to increased anxious/depressive symptoms as evidenced by numerous studies [1, 2, 34, 35]. Yet, many studies addressing this topic focused on the general population [1, 2] or individuals with dementia [4, 36]. Investigating the mental health implications of confinement in populations at elevated risk of cognitive decline can be instrumental to plan interventions and improve their clinical prognosis. In this regard, stress perception and stress resilience are especially interesting as they are shaped by individual factors or can be enhanced by training.

Herein, we focused on CU adults with a subgroup experiencing SCD, many of them being first-order descendants of sporadic AD patients and thus at a higher risk of developing cognitive decline and AD dementia [37, 38]. Our main aim was to investigate whether the intensity of SCD was associated with greater anxious/depressive symptoms during the COVID-19 confinement independent of pre-confinement anxiety/depression levels. Furthermore, we investigated the impact of brain Aβ status, stress perception, and stress resilience on these associations. Finally, we evaluated whether participants with or without SCD showed differences in worries and lifestyle changes during the confinement.

## Methods

### Participants and study design

Participants were selected from the longitudinal ALzheimer’s and FAmilies (ALFA+) study, nested to the ALFA parent cohort. ALFA parent cohort participants were recruited between 2013 and 2014 and included 2743 middle-aged (45–74 years) CU individuals, with the majority having a family history (FH) of sporadic AD [37]. The nested ALFA+ study included 451 cognitively intact participants (Clinical Dementia Rating = 0, Mini-Mental State Examination ≥ 27, semantic fluency ≥ 12) without any significant systematic illness, major psychiatric disorder, or unstable medical condition. ALFA+ participants underwent detailed cognitive testing, clinical interviews, lifestyle questionnaires, APOE genotyping, and blood and cerebrospinal fluid extractions, as well as magnetic resonance imaging and Aβ positron emission tomography (PET) data acquisitions [37]. These data were collected between 2016 and 2019 (average time lapse from ALFA+ HADS data acquisition to confinement data acquisition: 2.4 ± 0.8 years) from ALFA+ participants and referred to as “pre-confinement” measurements in the current study.

On May 8, 2020, during the COVID-19-related home confinement de-escalation periods in Spain [39], an invitation to participate in the current study was sent via an online link to 2582 participants of the ALFA parent cohort. Among these, 265 participants from the ALFA+ study responded positively to the invitation and completed an online assessment battery between May 8, 2020, and July 7, 2020, the period referred to as “confinement” in the present study. The assessment battery consisted of measurements of anxiety, depression, stress perception, stress resilience, and an ad hoc questionnaire developed to investigate specific worries and changes in lifestyle during the confinement. A total of 250 ALFA+ participants that completed the Hospital Anxiety and Depression Scale (HADS) in the assessment battery were included in the current study. As described below, participants had clinical data (SCD and HADS assessments) and Aβ PET data (*N* = 205) acquired during the pre-confinement period.

### Clinical assessments

#### SCD intensity and status

During the pre-confinement period (in the context of the ALFA+ study), participants filled out the MyCog part of the Subjective Cognitive Decline-Questionnaire (SCD-Q) [40] before neuropsychological testing. The SCD-Q measures the presence or absence of a self-perceived deterioration in cognitive performance in the last two years of daily life. The MyCog part consists of 24 questions inquiring about the subjective experience of difficulties in performing memory, language, and executive tasks. Here, we investigated the intensity of SCD using MyCog scores as a continuous measurement of SCD symptoms, with higher scores indicating greater complaints in cognitive performance [26, 40]. The rationale behind this approach was based on the characteristic of our sample (middle-aged adults) and in consequence the relatively low percentage of participants classified as SCD with a binary classification (yes/no). Nevertheless, to allow comparison with previous and future research and test the between-group differences, we also classified participants as having SCD if the answer to the first question “Do you perceive memory or cognitive difficulties?” was affirmative and without SCD if the answer was negative [41]. Finally, we performed a sensitivity analysis with participants classified as SCD aged 60 and above.

#### Anxiety and depression

We used the 14-item HADS composed of the 7-item anxiety and the 7-item depression subscale to measure the symptoms of anxiety/depression. In each subscale, the items are scored from 0 to 3, generating a total score between 0 and 21. Higher scores indicate a greater level of anxiety or depression. The sum of 14 items generates a total anxiety/depression score [42].

#### Stress perception and stress resilience

Participants completed the 10-item Perceived Stress Scale (PSS) [43] as a measurement of self-reported stress perception during the confinement. Scores in the PSS range from 0 to 40 with higher ones reflecting a greater perception of stress. Furthermore, we evaluated the participants’ ability to resist or recover from stress using the 6-item Brief Resilience Scale (BRS) [44]. Higher scores in BRS indicate higher resilience to stress.

#### Worries and lifestyle changes during the COVID-19 confinement

We developed an ad hoc questionnaire that focused on the worries about access to primary products, self-protection materials, and economic situation, as well as changes in sleep duration, sleep quality, and eating habits during the confinement. The questions and the procedure to dichotomize the answers are provided in Additional file 1.

### Aβ PET data acquisition and pre-processing

[^18^F] flutemetamol PET acquisitions were performed at the pre-confinement period in 205 participants in a Siemens Biograph mCT (Munich, Germany) following a cranial computed tomography scan for attenuation correction; 185 MBq (range 166.5–203.5 MBq) of [^18^F] flutemetamol was injected to the participants, and four frames of 5 min each were acquired 90 min post-injection. An OSEM3D algorithm with 8 iterations and 21 subsets was used for image reconstruction with point spread function and time-of-flight corrections into a matrix size of 1.02 × 1.02 × 2.03 mm.

All PET images were preprocessed using SPM12 (Statistical Parametric Mapping, Welcome Trust Centre for Neuroimaging, UK; http://www.fil.ion.ucl.ac.uk/spm). The averaged PET images were co-registered to the corresponding T1-weighted images. Then, T1-weighted images were segmented and normalized to Montreal Neurological Institute (MNI) space alongside the PET images. The standardized value uptake ratio (SUVR) was calculated in MNI space from the bilateral frontal and parietotemporal areas, and the whole cerebellum was used as a reference region. We then converted the SUVR values to the Centiloid (CL) [45] scale (see [46] for details). The cutoff value for CL was defined with a threshold of 12, and the scans were classified as “Aβ-negative” (< 12 CL) or “Aβ-positive” (≥ 12 CL) [46].

### Statistical analyses

Descriptive statistics were generated with means and standard deviations for continuous variables and percentages and frequencies for categorical variables. We performed chi-square analyses to explore the differences in worries and lifestyle changes during the COVID-19 confinement between participants classified as SCD or without SCD.

We conducted analyses of covariance (ANCOVA) with HADS scores during the confinement as the dependent variable, adjusted by pre-confinement HADS scores. Firstly, we tested whether higher SCD-Q MyCog scores were associated with higher anxiety/depression scores during the confinement independent of the pre-confinement anxiety/depression levels. We adjusted the model by age, sex, years of education, and the inter-individual time variability between pre-confinement and confinement assessments. In the second step, we introduced Aβ positivity as well as stress perception (PSS) and stress resilience (BRS) in the model. Finally, we performed mediation analyses using bootstrap procedures (computed for *N* = 1000 bootstrapped samples) to investigate whether the tested association between SCD-Q and HADS scores was mediated by Aβ positivity or stress-related measurements.

Statistical analyses were conducted with IBM SPSS Statistics for Windows (Build 1.0.0.1447, Armonk, NY), and for the mediation analysis, the R Statistical Software (version 4.0.2) “mediation” package was used (R Foundation for Statistical Computing, Vienna, Austria). The results yielding a *p* value lower than 0.05 were considered as statistically significant.

## Results

In the whole sample, participants’ age ranged from 52 to 72 years; 153 (61.2%) were women, and 248 (99.2%) were white Caucasians (0.8% Latinos). The average MyCog score was 4.1 (± 4.4) and 55 (22.3%) showed high-intensity SCD (MyCog scores ≥ 7) [47]. Seventy (28%) participants were classified as having SCD. A total of 197 participants (78.8%) and 53 of those classified as SCD (75.7%) had at least one parent diagnosed with sporadic AD. Twenty-six (12.7%) out of the 205 participants with available Aβ PET scans were Aβ-positive. Twenty-two (31.4%) of those classified as SCD sought medical help due to the perceived decline in cognitive abilities. During the confinement, 26 (10.4%) participants were diagnosed or under treatment for anxiety or depression. Table 1 displays the sociodemographic, clinical, and imaging data for the whole sample and participants classified as SCD vs. without SCD.

**Table 1:** Demographic, imaging, and clinical characteristics of the study participants

	Overall	With SCD	Without SCD
Variable	***N*** = 250	***N*** = 70	***N*** = 180
**Age, years**	63.5 (4.8)	63.9 (4.3)	63.3 (4.9)
**Female,*n*(%)**	153 (61.2)	43 (61.4)	110 (61.1)
**Family history of sporadic AD,*n*(%)**^a^	197 (89.1)	53 (75.7)	144 (80)
**Caucasian,*n*(%)**	248 (99.2)	68 (97.1)	180 (100)
**Education, years**	13.3 (3.5)	13.8 (3.4)	13.1 (3.5)
**Aβ**-**positive (≥ 12*CL*),*n*(%)**^b^	26 (12.7)	7 (10)	19 (10.6)
**SCD-Q, MyCog scores**^c^	4.1 (4.4)	8.2 (4.5)	2.5 (3.1)
**Pre-confinement HADS total scores**	6.6 (4.8)	8.3 (5.1)	6 (4.5)
**Confinement HADS total scores**	8.1 (5.9)	9.4 (6.1)	7.6 (5.7)
**PSS scores**^d^	16.1 (8.6)	18 (9.1)	15.4 (8.3)
**BRS scores**^e^	3.2 (0.4)	3.1 (0.3)	3.2 (0.4)
**Change in HADS from pre-confinement to confinement**	1.5 (5.8)	1.1 (5.2)	1.6 (5.9)
**Currently diagnosed/under treatment for anxiety/depression,*n*(%)**^f^	26 (10.4)	9 (12.9)	17 (9.4)
**Seeking medical help due to the self-perceived decline in cognition**	22 (8.8)	22 (31.4)	–

Data are presented as mean (standard deviation) unless otherwise indicated

*Abbreviations*: *AD* Alzheimer’s disease, *SCD* subjective cognitive decline, *Aβ* amyloid-beta, *CL* Centiloid, *SCD-Q* Subjective Cognitive Decline Questionnaire, *HADS* Hospital Anxiety and Depression Scale, *PSS* Perceived Stress Scale, *BRS* Brief Resilience Scale

^a^Overall *N* = 221

^b^Overall *N* = 205

^c^Overall *N* = 246

^d^Overall *N* = 238

^e^Overall *N* = 238

^f^Overall *N* = 248

Aβ burden (measured with continuous CL) did not show any correlation with PSS (*r* = 0.13; *p* = 0.078) or BRS (*r* = 0.044; *p* = 0.53) scores. We did not find a correlation between PSS and BRS scores during the confinement (*r* = 0.058; *p* = 0.37).

Participants having SCD showed significantly higher MyCog scores than those without SCD (*t*_95.2_=9.74; *p* < 0.001). Regarding anxiety/depression symptoms, we found a significant increase in HADS scores from pre-confinement to confinement (*t*_249_ = 4.04; *p* < 0.001) in the whole sample. Moreover, a lower frequency of going outside the home was associated with higher HADS scores during the confinement (*F* = 3.10; *p* = 0.017). This result suggests that the HADS evaluations are, at least partially, a reflection of the intensity of the COVID-19 confinement.

Participants with or without FH of sporadic AD did not present any difference in SCD-Q (*t*_26.6_ = − 1.51; *p* = 0.14) or HADS scores (pre-confinement: *t*_31.2_ = − 0.17; *p* = 0.87, during the confinement: *t*_34.2_ = 1.10; *p* = 0.29).

### Differences in SCD status in pre-confinement anxiety/depression measurements and confinement-related stress perception and stress resilience

Participants classified as having SCD had higher pre-confinement HADS scores (*p* < 0.001) than those without SCD. Regarding stress-related measurements, participants with SCD showed higher PSS scores (*t*_236_ = 2.12; *p* = 0.035) during the confinement compared to those without SCD. No differences were found in BRS scores between the two groups (*t*_246_ = − 0.45; *p* = 0.65).

In the whole sample, SCD-Q scores were positively correlated with pre-confinement HADS (*r* = 0.37; *p* < 0.001) and PSS (*r* = 0.43; *p* < 0.001) scores whereas they did not show any correlation with BRS scores (*r* = 0.044; *p* = 0.49).

### Differences in SCD status in worries and lifestyle changes during the COVID-19 confinement

**Fig. 1:**
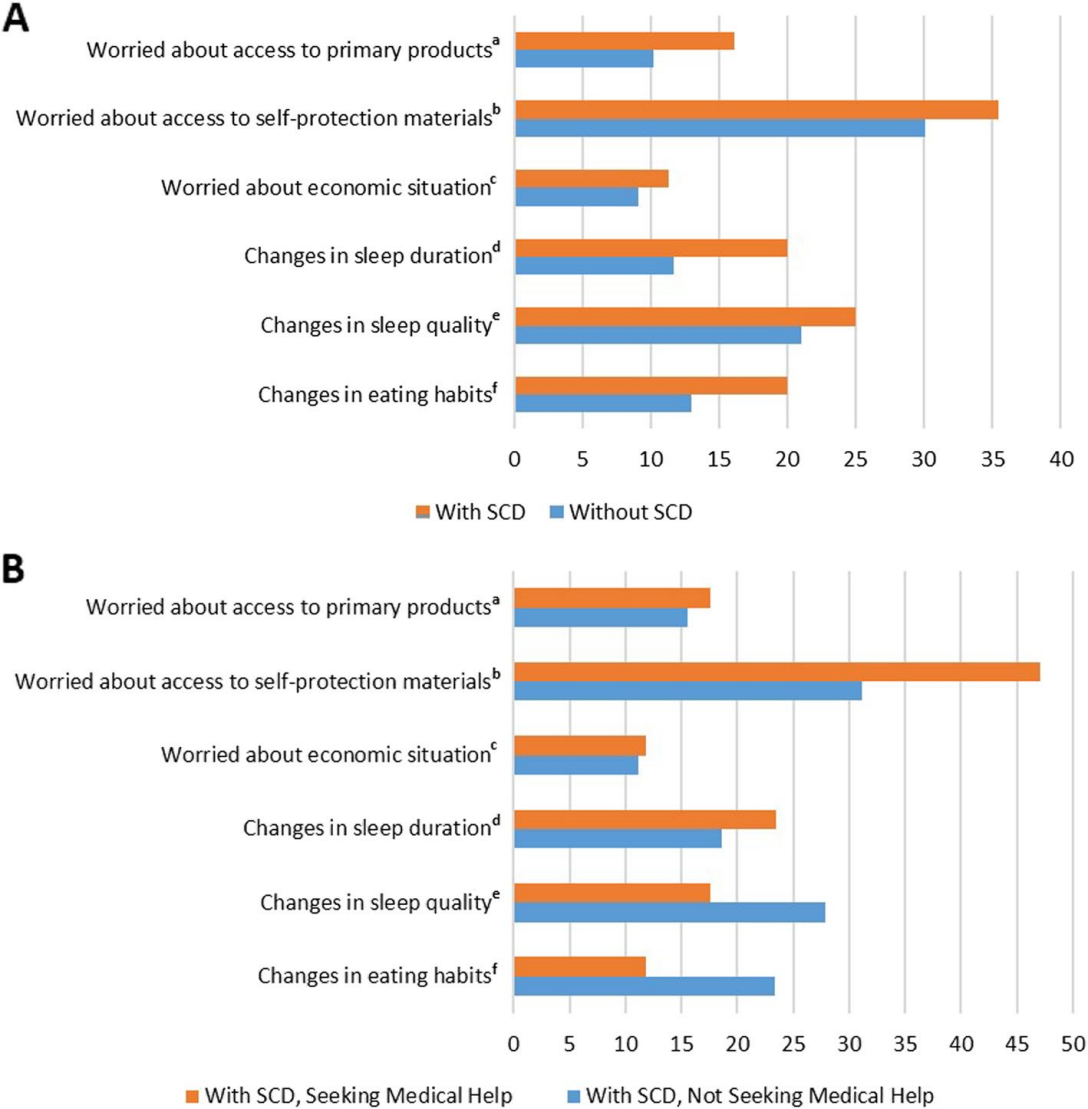
Confinement-related worries and lifestyle changes in the whole sample and in the subsample with SCD. **A** In the whole sample, the percentage of participants showing specific worries and lifestyle changes during the confinement is displayed by SCD status. ^a^With SCD *N* = 62, without SCD *N* = 167. ^b^With SCD *N* = 62, without SCD *N* = 166. ^c^With SCD *N* = 62, without SCD *N* = 165. ^d^With SCD *N* = 60, without SCD *N* = 162. ^e^With SCD *N* = 60, without SCD *N* = 162. ^f^With SCD *N* = 60, without SCD *N* = 162. **B** In the subsample with SCD, the percentage of participants showing specific worries and lifestyle changes during the confinement is displayed for the participants who sought medical help and the participants who did not seek medical help. ^a^With SCD, seeking medical help *N* = 17; with SCD, not seeking medical help *N* = 45. ^b^With SCD, seeking medical help *N* = 17; with SCD, not seeking medical help *N* = 45. ^c^With SCD, seeking medical help *N* = 17; with SCD, not seeking medical help *N* = 45. ^d^With SCD, seeking medical help *N* = 17; with SCD, not seeking medical help *N* = 43. ^e^With SCD, seeking medical help *N* = 17; with SCD, not seeking medical help *N* = 43. ^f^With SCD, seeking medical help *N* = 17; with SCD, not seeking medical help *N* = 43. *Abbreviations*: SCD, subjective cognitive decline

The results of the chi-square analyses investigating the differences between participants with and without SCD in confinement-related worries (access to primary products, self-protection materials, and economic situation) and lifestyle changes (sleep duration, sleep quality, and eating habits) are reported in Table 2. These analyses revealed that there were no significant differences between the two groups in any of the domains assessed (Fig. 1A).

**Table 2:** Results from the chi-square analysis investigating the difference in confinement-related worries and lifestyle changes by SCD status

	With SCD (%)		Without SCD (%)		***p*** value
	Yes	No	Yes	No	
**Worries**					
Access to primary products^a^	16.1	83.9	10.2	89.8	0.21
Access to self-protection materials^b^	35.5	64.5	30.1	69.9	0.44
Economic situation^c^	11.3	88.7	9.1	90.9	0.62
**Lifestyle changes**					
Changes in sleep duration^d^	20	80	11.7	88.3	0.11
Changes in sleep quality^e^	25	75	21	79	0.52
Changes in eating habits^f^	20	80	13	87	0.19

*Abbreviation*: *SCD* subjective cognitive decline

^a^With SCD *N* = 62, without SCD *N* = 167

^b^With SCD *N* = 62, without SCD *N* = 166

^c^With SCD *N* = 62, without SCD *N* = 165

^d^With SCD *N* = 60, without SCD *N* = 162

^e^With SCD *N* = 60, without SCD *N* = 162

^f^With SCD *N* = 60, without SCD *N* = 162

### Association between the intensity of SCD and anxiety/depression measurements

Higher SCD-Q MyCog scores were associated with greater HADS scores irrespective of the pre-confinement anxiety/depression levels. Furthermore, younger age, lower years of education, and having higher pre-confinement HADS scores showed independent associations with greater HADS scores during the confinement (Table 3).

**Table 3:** Results from the model showing the association of SCD-Q with anxiety/depression scores

Predictor	Confinement HADS	
	***B*** value (95% CI)	***p*** value
SCD-Q, MyCog scores	0.25 (0.096 to 0.41)	**0.002**
Age, years	− 0.19 (− 0.33 to − 0.055)	**0.006**
Sex (women)	1.25 (− 0.12 to 2.63)	0.074
Education, years	− 0.31 (− 0.50 to − 0.12)	**0.001**
Pre-confinement HADS	0.42 (0.27 to 0.56)	**< 0.001**
Time difference	0.38 (− 0.26 to 1.03)	0.240

The unstandardized *B* represents the variation in confinement HADS scores with 1-unit variation in a given predictor

*Abbreviations*: *HADS* Hospital Anxiety and Depression Scale, *CI* confidence interval, *SCD-Q* Subjective Cognitive Decline Questionnaire

### Association between the intensity of SCD and anxiety/depression measurements: model including Aβ status and stress-related variables

Following the inclusion of Aβ positivity, PSS, and BRS in the model, SCD-Q MyCog scores did not show a significant association with HADS scores. Aβ positivity and PSS scores, instead, showed significant associations with higher HADS scores irrespective of the pre-confinement anxiety/depression levels. BRS scores were not associated with HADS scores. Additionally, sex (women) was associated with presenting greater HADS scores during the confinement (Table 4).

**Table 4:** Results from the model including Aβ positivity and stress-related measurements

Predictor	Confinement HADS	
	***B*** value (95% CI)	***p*** value
SCD-Q, MyCog scores	0.12 (− 0.01 to 0.25)	0.069
Aβ positivity	1.95 (0.28 to 3.63)	**0.023**
PSS scores	0.49 (0.43 to 0.56)	**< 0.001**
BRS scores	− 0.28 (− 1.56 to 1.01)	0.670
Age, years	− 0.11 (− 0.24 to 0.008)	0.068
Sex (women)	1.11 (0.006 to 2.26)	**0.049**
Education, years	− 0.12 (− 0.28 to 0.033)	0.120
Pre-confinement HADS	0.01 (0.11 to 0.13)	0.870
Time difference	0.4 (− 0.17 to 0.98)	0.170

The unstandardized *B* represents the variation in confinement HADS scores with 1-unit variation in a given predictor

*Abbreviations*: *HADS* Hospital Anxiety and Depression Scale, *CI* confidence interval, *Aβ* amyloid-beta, *SCD-Q* Subjective Cognitive Decline Questionnaire, *PSS* Perceived Stress Scale, *BRS* Brief Resilience Scale

### Mediation analyses

**Fig. 2:**
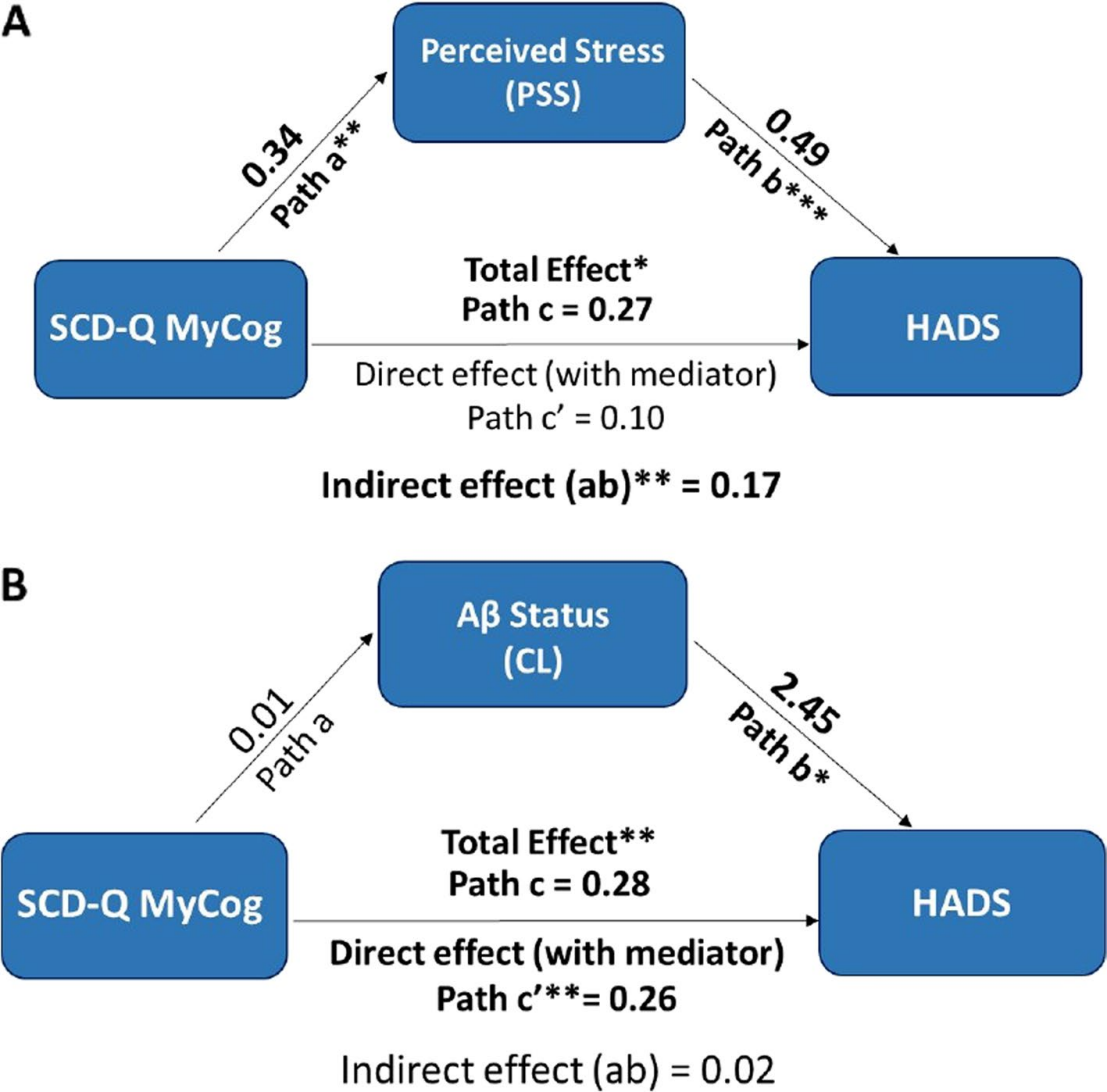
Schematic illustration of the mediation analyses investigating the link between SCD and anxiety/depression scores. Regression coefficients for paths a, b, c, c′, and ab are reported in the figure. The total effect of SCD-Q MyCog on HADS (*path c*) is shown as the summation of the direct effect adjusted by the given mediator (*c′*) and the indirect effect (*ab*). The analyses were adjusted by age, sex, years of education, pre-confinement HADS scores, and time difference between the pre-confinement and confinement assessments. *p < 0.05, **p < 0.01, ***p < 0.001. **A** The results of the mediation analysis investigating the role of PSS as a mediator between SCD-Q MyCog scores and HADS scores. The indirect effect (path ab) was statistically significant (*p* = 0.01) corresponding to 63% of the total association between the two variables. **B** The results of the mediation analysis investigating the role of Aβ as a mediator between SCD-Q MyCog and HADS scores. The indirect effect (path ab) did not show a statistically significant effect (*p* = 0.45). *Abbreviations*: SCD-Q, Subjective Cognitive Decline Questionnaire; PSS, Perceived Stress Scale; HADS, Hospital Anxiety and Depression Scale; Aβ, amyloid-beta; CL, Centiloid

SCD-Q MyCog scores did not show a significant association with HADS scores after including Aβ status and stress-related variables in the model. Since both Aβ positivity and PSS were associated with HADS scores, we performed mediation analyses to investigate whether the effect of SCD-Q on anxiety/depression scores was mediated by Aβ positivity and/or stress perception. As illustrated in Fig. 2A, we found a significant partial mediation effect of stress perception on the association of SCD-Q MyCog scores with confinement HADS scores corresponding to 63% of the total association between the two variables (*B* = 0.17, 95% CI = 0.03 to 0.31, *p* = 0.01). Amyloid-β positivity, instead, did not show a mediating role between SCD-Q MyCog and HADS scores (*B* = 0.02, 95% CI = − 0.01 to 0.06, *p* = 0.45, respectively, see Fig. 2B).

### Sensitivity analyses

#### Models with SCD status

We repeated our main statistical analysis using the dichotomous SCD variable (participants classified as having SCD vs. without SCD) that is often used in the SCD field and in clinical practice [41]. The results showed that, unlike continuous SCD-Q scores, SCD status did not show an association with HADS scores during the confinement (95% CI = − 0.38 to 2.58, *B* = 1.10, *p* = 0.14).

#### SCD plus criteria-persistent SCD and age at onset of SCD after 60 years old

Given that the SCD-Q and SCD classification was performed based on pre-confinement evaluations, we repeated our main analysis with those participants that have a follow-up evaluation (*N* = 219) and were classified as SCD (*N* = 39). Persistent SCD (classified as SCD in both time points) was associated with greater HADS scores irrespective of pre-confinement HADS scores (95% CI = 0.07 to 3.73, *B* = 1.90, *p* = 0.042).

In addition, we repeated our models excluding those participants classified as SCD and aged below 60 (*N* = 27). The main results remained unchanged: the categorical SCD variable did not show a significant association with HADS scores (95% CI = − 0.15 to 3.66, *B* = 1.75, *p* = 0.071). Higher SCD-Q scores, instead, were associated with confinement HADS scores irrespective of pre-confinement anxiety/depression levels (95% CI = 0.13 to 0.52, *B* = 0.32, *p* = 0.001).

#### Differences in worries and lifestyle changes between participants with SCD that sought medical help vs. those that did not seek medical help

Within the group of participants classified as having SCD, we investigated whether those that sought medical help due to the perceived decline in cognition showed any differences in confinement-related worries and lifestyle changes than the ones that did not seek medical help (Fig. 1B). The rationale behind this sensitivity analysis is that seeking medical help is linked to worrying about the subjective decline in cognition [12, 47]. The two groups did not show any differences in worries about access to primary products (*X*^2^(1) = 0.04; *p* = 0.84), self-protection materials (*X*^2^(1) = 1.37; *p* = 0.24), or economic situation (*X*^2^(1) = 0.005; *p* = 0.94). We also did not observe any differences between the groups in changes in sleep duration (*X*^2^(1) = 0.18; *p* = 0.67), sleep quality (*X*^2^(1) = 0.68; *p* = 0.41), or eating habits (*X*^2^(1) = 1.005; *p* = 0.32) during the confinement.

#### Models adjusted by the change in sleep duration

We adjusted our main models by the change in sleep duration during the confinement. Following this adjustment, previously reported associations between SCD-Q and HADS scores did not change. Additionally, change in sleep duration showed a significant association with confinement HADS scores irrespective of pre-confinement anxiety/depression levels (95% CI = 1.49 to 5.35, *B* = 3.42, *p* < 0.001).

## Discussion

In the current study, we investigated the association of the intensity of SCD with anxious/depressive symptoms during the COVID-19-related home confinement in a cohort of CU adults with an elevated risk of developing AD dementia. Our findings showed that higher levels of SCD, as measured by MyCog scores, were associated with presenting greater anxious/depressive symptoms during the COVID-19 confinement, irrespective of the pre-confinement anxiety/depression levels. Furthermore, Aβ positivity and higher stress perception showed independent associations with greater anxiety/depression scores. Mediation analyses revealed that the association of SCD intensity with higher anxiety/depression symptoms was mediated by perceived stress but not Aβ positivity. Lastly, we did not observe any differences in worries or lifestyle changes between the participants with or without SCD during the confinement.

During the COVID-19 pandemic, severe restrictions implemented to prevent the spread of the disease had negative effects on people’s mood, lifestyle, and mental health [33, 48]. In line with previous reports [49, 50], our sample showed increases in anxious/depressive symptoms between pre-confinement and confinement measurements. In addition, although the majority of the participants were not diagnosed with anxiety or depression during the confinement, the observed increase in anxiety/depression scores from pre-confinement (1.5 ± 5.8) was within the range of the minimum clinically significant difference suggested for the HADS [51, 52]. While participants having SCD had higher pre-confinement and confinement anxiety/depression scores than the ones without SCD, only the latter showed a minimum clinically significant difference from pre-confinement to confinement (mean change: 1.6 ± 5.9). Previous studies suggested an association of FH of dementia with greater cognitive complaints [53, 54] which could be mediated by depressive symptoms [55]. However, we did not observe any significant differences in HADS in those with and without FH of sporadic AD in our sample. Furthermore, having higher pre-confinement HADS scores independently contributed to explain higher HADS scores during the confinement. These results suggest that home confinement had a global influence on anxious/depressive symptomatology, including in individuals without cognitive complaints [2, 7, 36].

In line with our hypothesis, participants having higher levels of SCD showed higher anxious/depressive symptoms during the confinement irrespective of the pre-confinement anxiety/depression levels. These results are consistent with previous studies showing associations between SCD and affective symptoms [26, 56], specifically during the pandemic [3, 6, 7]. Previous studies performed during the pandemic focused on the differences in mental health outcomes between participants with SCD [7] and with mild cognitive impairment (MCI) or AD dementia [3, 6]. A strength of our study, compared to previous work, is that our cohort consisted of CU participants with a subgroup having SCD that allowed us to evaluate the differences in anxious/depressive symptoms between participants without and with SCD considering the intensity of the symptoms. When the categorical SCD variable was considered, having SCD did not show an association with anxiety/depression symptoms. When we performed the analysis using a longitudinal SCD classification (i.e., persistency of SCD over time), persistent SCD status was associated with HADS scores independent of pre-confinement HADS levels. The difference in the results depending on the approach used for SCD could reflect a limitation of using a single general question to determine the SCD status on a single time point. Considering that our main findings are consistent with the previous studies using SCD-Q MyCog scores as a continuous spectrum of SCD-related complaints [26, 40], these results suggest that the intensity of SCD could be a more sensitive approach to capture associations with anxiety/depression in middle-aged adults when only one time point assessment is available.

Our results showed that Aβ positivity measured 2.4 years before the confinement was associated with greater anxious/depressive symptoms during the confinement. These findings are consistent with the literature reporting associations between Aβ pathology and neuropsychiatric symptomatology in cognitively unimpaired older adults [19, 21–23], although the direction of causality between the two events is not clear. Previous studies suggested that anxious/depressive symptoms might facilitate or accelerate the expansion of Aβ and pathological progression of the disease [57, 58]. Conversely, specific neuropsychiatric symptoms, such as anxiety and depression, may represent an early manifestation of AD pathophysiology [23, 59]. Longitudinal studies are required to validate this hypothesis and unravel the potential causal relationship between the two events.

Higher levels of stress are often associated with negative emotions [60] and the development of affective symptoms, in particular during negative life events [61, 62]. The studies conducted during the pandemic suggested that confinement acts as the main stressor having an adverse impact on mental health [33, 35]. In our sample, we found that the association of higher levels of SCD with anxious/depressive symptoms was mediated by self-perceived stress during the confinement. This result is consistent with the reported associations between subjective cognitive complaints and stress perception in older adults [25, 33, 63]. The dysregulation in the hypothalamic-pituitary-adrenal axis may explain these associations such that stress-induced glucocorticoid secretion could have detrimental effects on memory performance [64, 65]. In agreement with these findings, our results suggest that people with higher levels of SCD are more vulnerable to stressors which may hasten the development of negative mental health outcomes. Overall, these findings support the previous research suggesting that interventions targeting stress alleviation can improve mental health outcomes in older adults with SCD [66].

Recent studies observed COVID-19 pandemic-related worries (e.g., related to getting the COVID-19 disease or socio-economic concerns) and lifestyle changes (e.g., changes in sleep or eating habits) in the general population [35, 48, 67] as well as in adults with SCD, MCI, or dementia [3, 6, 7]. We investigated whether the participants classified as SCD presented greater levels of worries and lifestyle changes during the confinement compared to those without SCD. Contrary to our hypothesis, the two groups did not show any differences in worries related to access to primary products, self-protection materials, or economic situation during the confinement. One possible explanation could be that the higher levels of worries observed in individuals with SCD [68] may be specific to cognitive decline-related concerns [12, 47] rather than reflecting a general trait of worrying in this population. However, our assessment did not include worries about the cognitive status during the confinement as in recent studies [3, 69]. Similarly, we did not observe significant differences in changes in sleep duration, sleep quality, or eating habits between the two groups. Previous studies found lower sleep duration [70] or sleep quality [71] in adults with SCD as compared to those without SCD using objective measurements. However, our questionnaire investigating the lifestyle changes was self-reported which could possibly explain the different results.

Lastly, we observed higher anxious/depressive symptoms during the COVID-19 confinement in younger participants and those with fewer years of education. Following the inclusion of Aβ positivity and stress-related measurements, sex (women) also showed an association with higher anxiety/depression scores. Overall, these results are in line with recent studies showing a higher prevalence of mental health problems in women, younger aged, and lower educated adults during the COVID-19 pandemic [2, 50, 72, 73]. Many studies attributed these findings to financial inequalities, poor economic status, and unemployment that have been shown as risk factors for developing anxiety and depression during the pandemic, particularly in these groups [2, 72]. Moreover, economic uncertainties and associated distress may exacerbate the negative consequences of the pandemic on the mental well-being of these populations in the long term [2, 74].

## Limitations

Our study has several limitations. Firstly, our main results are reported on individuals recruited for a research study, and the majority have a FH of sporadic AD, linked previously with greater levels of cognitive complaints [53–55]. However, we did not observe any difference in SCD intensity between participants with or without FH of AD in our sample. Nevertheless, extrapolation to the general SCD population should not be done without further research. Secondly, among the ALFA+ participants that received the invitation to participate in the current study, only 59% of them responded. However, there were no significant differences in demographics or pre-confinement HADS scores between the responders and non-responders (data not shown). Furthermore, although the measurements of anxiety/depression were collected during the COVID-19-related home confinement, we cannot directly attribute the reported anxious/depressive symptoms in SCD to the confinement. Neuropsychiatric symptoms may also appear as part of the preclinical AD process [23, 59]. However, our HADS measurements were associated with the intensity of the confinement. In the same vein, the clinical measurements used in this study were self-reported, and objective measurements may provide different results. Yet, our results are supported by previous studies reporting higher anxiety, depression, or stress in similar cohorts during the confinement using subjective scales [3, 33]. Finally, our participants performed the SCD-Q before the pandemic, and SCD was not assessed during the COVID-19 confinement. Therefore, possible changes in the cognitive status of the participants from pre-confinement to confinement were not evaluated. However, with the available data collected after the confinement, we were able to repeat the main analysis only in the subsample that showed persistent SCD (*N* =39), and the results remained the same.

## Conclusions

Our findings showed that adults at increased risk of AD and with higher levels of SCD are more vulnerable to adverse mental health outcomes during the COVID-19-related confinement. Furthermore, the mental health burden observed in adults with SCD may be mediated by stress perception. These results emphasize the importance of investigating the role of stress management in reducing the affective symptoms in the SCD population that could potentially lower the overall risk of cognitive impairment and dementia in the long term.

## Supplementary Information

**Additional file 1: Table S1**. Selected questions from the online ad-hoc questionnaire on worries and lifestyle changes during the COVID-19 confinement.

## Data Availability

The data supporting the results of the current study are not publically available but may be available upon a reasonable request.

## Abbreviations

Aβ: Amyloid-beta
AD: Alzheimer’s disease
ALFA: ALzheimer’s and FAmilies
BRS: Brief Resilience Scale
CL: Centiloid
FH: Family history
HADS: Hospital Anxiety and Depression Scale
PET: Positron emission tomography
PSS: Perceived Stress Scale
SCD: Subjective cognitive decline
SCD-Q: Subjective Cognitive Decline Questionnaire
