# Supplementary material for: Subjective cognitive decline and anxious/depressive symptoms during the COVID-19 pandemic: what is the role of stress perception, stress resilience, and β-amyloid?
Source: Alzheimers Res Ther. 2022 Sep 6;14:126. doi: 10.1186/s13195-022-01068-7 (PMC9446623; doi:10.1186/s13195-022-01068-7)
Supplement: Supplementary file 1 — Additional file 1: Table S1. Selected questions from the online ad-hoc questionnaire on worries and lifestyle changes during the COVID-19 confinement. [file 13195_2022_1068_MOESM1_ESM.docx]

**Additional File 1 Selected questions from the online *ad-hoc* questionnaire on worries and lifestyle changes during the COVID-19 confinement**

| **Questions** | **Answers** | |
| --- | --- | --- |
| **Worries** |  | |
| **Worries about access to primary products:^a^**  During confinement, are you concerned or have you ever been concerned about having access to first need products? | Yes | No |
| **Worries about access to self-protection materials:^a^**  Are you concerned about having access to material you need to protect yourself, such as face masks? | Yes | No |
| **Worries about the economic situation:^b^**  Are you currently concerned about your present or future economic situation due to the crisis produced by the pandemic? | Yes | No |
| **Lifestyle Changes** |  | |
| **Changes in sleep duration:**  I cannot maintain my usual sleeping habits (I sleep at odd hours, less or more than normal). | Yes | No |
| **Changes in sleep quality:**  I cannot sleep well (It is difficult for me to fall asleep, I wake up several times at night). | Yes | No |
| **Changes in eating habits:**  I cannot eat at least two meals a day or I cannot avoid overeating. | Yes | No |

a: The following responses were dichotomized under “No” category: *(i) No, I have never been worried, (ii) No I am not worried, but I was worried at the beginning of the confinement.* The following responses were dichotomized under “Yes” category*: (iii) Yes, but I always have had access to everything I have needed, (iv) Yes, because I have not always had access to everything I have needed*. b: The following responses were dichotomized under “No” category: *(i) Never been worried, (ii) A bit worried, (iii) Having some worries.* The following responses were dichotomized under “Yes” category: *(iv*) *Quite worried*, *(v)* *Very worried*.
